# ﻿When the absence of evidence is not the evidence of absence: *Nasa* (Loasaceae) rediscoveries from Peru and Ecuador, and the contribution of community science networks

**DOI:** 10.3897/phytokeys.229.100082

**Published:** 2023-06-30

**Authors:** Tilo Henning, Rafael Acuña-Castillo, Xavier Cornejo, Paúl Gonzáles, Edgar Segovia, Akira Armando Wong Sato, Maximilian Weigend

**Affiliations:** 1 Leibniz Centre for Agricultural Landscape Research (ZALF), Eberswalder Str. 84, 15374, Müncheberg, Germany; 2 Escuela de Biología, Universidad de Costa Rica, Apdo. Postal 11501-2060, San Pedro de Montes de Oca, San José, Costa Rica; 3 Herbario Luis A. Fournier Origgi, Centro de Investigación en Biodiversidad y Ecología Tropical (CIBET), Universidad de Costa Rica, Apdo. Postal 11501–2060, San Pedro de Montes de Oca, San José, Costa Rica; 4 Herbario GUAY, Departamento de Botánica, Facultad de Ciencias Naturales, Universidad de Guayaquil, P.O. Box 09-01-10634, Guayaquil, Ecuador; 5 Laboratorio de Florística, Departamento de Dicotiledóneas, Museo de Historia Natural, Universidad Nacional Mayor de San Marcos, Av. Arenales 1256, Jesús María, Peru; 6 Universidad Católica de Cuenca, CIITT, 42VM+PJ4, Ricaurte, Azuay, Ecuador; 7 Plant Ecology Division, CORBIDI, Calle Santa Rita 105 Of. 2, Urb. Huertos de San Antonio Monterrico, Lima, Surco, Peru; 8 Nees-Institut für Biodiversität der Pflanzen, Universität Bonn, Meckenheimer Allee 171, 53115, Bonn, Germany

**Keywords:** Andes, conservation, iNaturalist, specimen, threatened species

## Abstract

Documentation of plant taxa has long been subject to the temporal and spatial selectivity of professional research expeditions, especially in tropical regions. Therefore, rare and/or narrowly endemic species are sometimes known only from very few and very old herbarium specimens. However, these taxa are very important from a conservation perspective. The lack of observations of living plants and confirmation of the actual occurrence of taxa hinders the planning and implementation of effective conservation measures. Community science networks have recently made tremendous contributions to documenting biodiversity in many regions across the globe. The rediscovery of six species of *Nasa* (Loasaceae) from Peru and Ecuador primarily via the platform iNaturalist, is reported.

## ﻿Introduction

In the past, taxonomic work was almost exclusively based on physical herbarium specimens. Herbarium specimens often lay undisturbed for decades or centuries, depending on the off chance of a specialist revising the holdings of a given collection (e.g., Cornejo 2017). Digitisation of specimens has dramatically improved access to herbarium collections, and nowadays specimens deposited in a herbarium may be accessible directly via the internet, rendering the comparison to type specimens dramatically easier than when physical access was still required (Hedrick et al. 2020). But this still means that a scientist with the necessary equipment and the required research and collection permits, has to encounter the plant in the wild, spend time and resources preparing a specimen and deposit it in a public repository, which then might be digitised at some stage. Much information is lost in the process, of course, and depending on the details recorded on the herbarium label, characters such as plant size or flower colour may be imperfectly captured. Herbarium collections remain an invaluable resource for taxonomic, floristic and systematic studies, but – by their very nature – reflect the actual distribution patterns imperfectly with a tremendous time lag (Bebber et al. 2010). If a species has gone uncollected for decades, especially in areas modified by human activities, specimen-based studies will suggest the species is extinct (Wood 2007; De Lírio et al. 2018; Pitman et al. 2022). This assumption could be reinforced if field searches of localities on herbarium labels fail to locate the species in its former habitat (Weigend 2000; Weigend and Rodríguez 2003).

The mostly tropical Andean genus *Nasa* Weigend is particularly relevant in this context: Due to its urticant nature and soft, quickly degrading leaves, the plants are difficult to collect. Additionally, the species of this genus tend to be rare, narrowly endemic, and highly seasonal – further reducing the likelihood of herbarium documentation. The genus as such is very widely distributed in tropical America from Veracruz (Mexico) to Antofagasta (Chile) on the western side of the Andes and Santa Cruz (Bolivia) in the east (Weigend 2001). However, *Nasa* reaches its highest diversity in the Amotape-Huancabamba Zone (AHZ), a region that encompasses southern Ecuador and northern Peru (Weigend 2002). In this phytogeographical zone, the highest diversity and density of taxa per unit of area is found and also the most range-restricted taxa (Mutke et al. 2014), some of which are endemic to a single known locality such as a single mountain summit or forest fragment (e.g., *Nasaglabra* (Weigend) Weigend, *N.kuelapensis* Weigend, *N.laxa* (J.F.Macbr.) Weigend, *N.pongalamesa* Weigend, *N.sanagoranensis* T.Henning, Weigend & A.Cano, *N.urentivelutina* Weigend). Further north or south of the AHZ, the distribution ranges of the taxa tend to cover larger areas, and thus, these species are less likely to be under threat (Mutke et al. 2014). On the other hand, many of the species that are restricted to the AHZ can be considered under some threat category according to national red list assessments (Rodríguez and Weigend 2006; Cornejo and Suin 2011).

In the course of systematic studies on predominantly Neotropical Loasaceae during the last decades, dozens of taxa previously unrecognized by science have been described. *Nasa* is the largest, most species-rich genus in the family (Weigend et al. 2006), with 55 of the 97 species and 21 subspecies described in the last ca. 25 years (e.g. Henning and Weigend 2011; Henning et al. 2011; Henning et al. 2019). Many of the species described during the 19^th^ and the first half of the 20^th^ century remained enigmatic – only known from the type collection or from a very limited number of often poorly preserved specimens. Some of these taxa, such as *Nasaaspiazui* (J.F.Macbr.) Weigend, *Nasamodesta* Weigend, *Nasapanamensis* Weigend, Nasarugosa(Killip)Weigendsubsp.rugosa, and *Nasarufipila* Weigend have highly distinctive characters, but for decades no new specimens have been included in public collections. Despite targeted field work by the authors and overall growing collection activities in the Neotropics, the species seem to have vanished from the localities where they were originally collected. Now, however, some of those taxa have recently been rediscovered, *Nasacolanii* Dostert & Weigend, *N.hastata* (Killip) Weigend and *N.solaria* (J.F. Macbr.) Weigend from Peru, *N.ferox* Weigend, the typical subspecies of *Nasahumboldtiana* (Urb. & Gilg) Weigend and *N.ramirezii* (Weigend) Weigend from Ecuador.

Different factors have lately come into play that resulted in these surprising rediscoveries that are summarised in this article. With the ongoing infrastructural development of the Andean countries, many new roads have increased accessibility, even to very remote areas and remaining habitat fragments (Torres Trujillo 2016; Corporación Andina de Fomento 2020). National research activities are steadily increasing in scope and efficiency. In addition, there is a vibrant scene of botanists, naturalists, environmentalists and hobbyists in, e.g., Peru and Ecuador, with a growing interest in nature and biodiversity and nature tourism, both by national and international citizens, and there has been a constant growth trend in the last decade (Santiago Chávez et al. 2017; Daries et al. 2021). Most importantly, it is no longer only professional botanists preparing herbarium specimens contributing to our understanding of biodiversity. Global networking and the increasing use of free data repositories and biodiversity networks have tremendously facilitated the presentation and availability of valuable data such as geo-referenced occurrence records and photos. iNaturalist is a particularly valuable platform for the exchange of (photographic) occurrence records. It is now considered one of the most influential community science projects (Aristeidou et al. 2021) and has already contributed towards the identification, location and description of previously unrecognised species (Winterton 2020; Alvarado Cárdenas et al. 2021). In addition to professional scientific collaborations for *N.colanii* and *N.humboldtiana*, a range of rediscoveries reported here come from fellow users of iNaturalist sharing their field images to discuss them with others.

The combination of recent field studies and a revision of digital data repositories considerably expands our understanding of the distribution patterns and status of several rare and/or putatively extinct taxa in *Nasa*.

## ﻿Materials and methods

The data for the five species included in this study were obtained from field trips, the iNaturalist.org platform, various literature references (see next sections) and material deposited at BM, E, F, GH, GOET, GUAY, HA, K, M, MO, MOL, OXF, P, S, US, USM and W. The type material and the protologues of all the species included here were examined. TH, RAC and MW have used iNaturalist to make their own field observations available to the scientific community and began curating other observations in their field of expertise. Four of the six taxa were rediscovered in this way: *Nasaferox*, *N.hastata*, *N.ramirezii* and *N.solaria*. In the case of the two other species, *Nasacolanii* and *N.humboldtiana*, the fellow scientists AAWS from Lima, Peru and XC from Guayaquil, Ecuador directly approached TH, RAC and MW in order to help/confirm their identifications. Data for *N.colanii* has subsequently been uploaded to iNaturalist.

Field trips in Ecuador and Peru were conducted to:

- The montane forest remnant at El Corazón (2°03'S, 78°54'W), 2500–2800 m (3167 m on google earth), in the province of Chimborazo, western Andes of Ecuador, during the months of July 2021 and August 2022, by XC.
- The NW buffer zone of El Cajas National Park (2°46'51.5"S, 79°15'56.6"W), 3826–3835 m, the province of Azuay, western Andes of Ecuador, during July 2021. *Nasa* specimens grew on a 30° inclined, NW oriented slope, on a rocky outcrop at the foot of a naked rocky peak, in the valley of the Río Cajas. Vegetation among the rocks was dominated by *Polylepisreticulata* Hieron. (Rosaceae), *Gynoxysminiphylla* Cuatrec. (Asteraceae), *Gynoxyscuicochensis* Cuatrec. (Asteraceae), and another indetermined Asteraceae, and a thick layer of bryophytes covered the ground. This habitat was surrounded by grasslands and disturbed habitat (used for llama ranching). The average daytime temperature at this locality was 13 °C, nighttime temperatures averaged < 0 °C and monthly precipitations varied from 35 mm in August, to 157 mm in March.
- The buffer zone of the Cordillera de Colán National Sanctuary (5°37'50.96"S, 78°15'20.84"W), 2600 m (1715 m on google earth), near the “Refugio Lechucita” of the Cordillera de Colán, in the department Amazonas, Peru, during September and December 2019 by AAWS. Rainfall is abundant and constant throughout the year with monthly averages of 91.9–226.8 mm, the forest is located on a slope oriented NE to SW. The months with the least rainfall are September, October and November, when the highest temperatures occur.
- The relict forest of Zárate (11°55'46.25"S, 76°29'36.55"W), 1400–3550 m, in the department Lima, Peru, in April 2009 by PG. Zárate has an average annual temperature of 12 °C and annual average precipitation of 360 mm, the forest is located on a slope oriented S and W, inclined between 45 and 90°, dominated by *Oreopanaxoroyanus* Harms (Araliaceae), *Myrcianthesquinqueloba* (McVaugh) McVaugh (Myrtaceae), *Escalloniaresinosa* Pers. (Escalloniaceae) and *Prunusrigida* Koehne (Rosaceae).
- The Arahuay village (11°37'17"S, 76°40'15"W), 2450 m (2495 m on google earth), department Lima, Peru, in April 2011 by PG. Arahuay has an average annual temperature of between 5–20 °C and annual average precipitation of 800 mm, and its shrubland is located on a slope oriented W, inclined between 20 and 45°, dominated by *Alonsoameridionalis* Druce (Scrophulariaceae), *Ambrosiaarborescens* Mill. (Asteraceae), *Baccharissternbergiana* Steud. (Asteraceae), *Calceolariaangustiflora* Ruiz & Pav. (Calceolariaceae), *Mutisiaacuminata* Ruiz & Pav. (Asteraceae), *Ophryosporusperuvianus* (J.F.Gmel.) R.M.King & H.Rob. (Asteraceae) and *Vasconcelleacandicans* A.DC. (Caricaceae)
- The Santa Rosa de Quives district (11°34'13.05"S, 76°42'12.23"W), 2450 m (2100 m on google earth), department Lima, Peru, in June 2012 by PG. Santa Rosa de Quives has an average annual temperature between 13–20 °C and annual average precipitation of 400 mm. The shrubland is located on a slope oriented S, inclined between 30 and 50°, dominated by *Barnadesiadombeyana* Less. (Asteraceae), *Chionopappusbenthamii* S.F.Blake (Asteraceae), *Jungiapauciflora* Rusby (Asteraceae), *Paracaliajungioides* (Hook. & Arn.) Cuatrec. (Asteraceae), *Lomanthuscantensis* (Cabrera) P.Gonzáles (Asteraceae), *Calceolaria angustiflora and Escallonia resinosa*.
- The relict forest at Huarimayo (11°30'29.59"S, 76°42'35.98"W), 2800–3000 m, department Lima, Peru, in May 2015 and May 2022, by PG. Huarimayo. It has an average annual temperature of 12 °C and annual average precipitation of 350 mm; the forest is located on a slope oriented SW, inclined between 45 and 90°, dominated by *Oreopanaxoroyanus*, *Myrcianthesquinqueloba*, *Escalloniaresinosa*, *Prunus rigida and Cervantesia bicolor* Cav. (Santalaceae).
- The archaeological complex of Rupac (11°19'23.00"S, 76°46'52.97"W), 3033–3099 m, department Lima, Peru, in April and May 2018. Rupac has an average annual temperature of 12 °C and annual average precipitation of 500 mm. Its shrubland is located on a slope oriented W, inclined between 20 and 60°, dominated by *Alonsoameridionalis*, *Calceolariaangustiflora*, *Lomanthussubcandidus* (A.Gray) B.Nord. (Asteraceae), *Mutisiaacuminata*, *Siphocampylustupaeformis* Zahlbr. (Campanulaceae), and *Vasconcelleacandicans*.

## ﻿Results

### ﻿ Nasa colanii

Taxon classificationPlantaeCornalesLoasaceae

Dostert & Weigend, Revista Peru. Biol. 13(1): 73 (2006).

93F3C9F7-0083-5DFF-85A4-513A06C1D00C

Fig. 1A, B

#### Type.

Peru. Amazonas: Provincia Bagua, Cordillera Colán SE of La Peca, ca. 3000 m, 25 Sep 1978, *P. Barbour 3573* (holotype: MO! [acc. # 2796329]; isotype: USM [acc. # 000462]).

The *Nasatriphylla*-group also includes two subscandent taxa with reflexed trichomes from montane rainforest, namely *Nasaaequatoriana* (Urb. & Gilg) Weigend and *Nasacolanii*. *Nasaaequatoriana* is well documented from Ecuador (Weigend 2000), while *Nasacolanii* was known only from a single Peruvian collection from 1978 (Dostert and Weigend 1999; Croat et al. 2021). *Nasacolanii* is probably the one species reported from the most inaccessible region of all the species here discussed – the Cordillera de Colán in northern Peru, near to the Ecuadorean Border.

**Figure 1.**
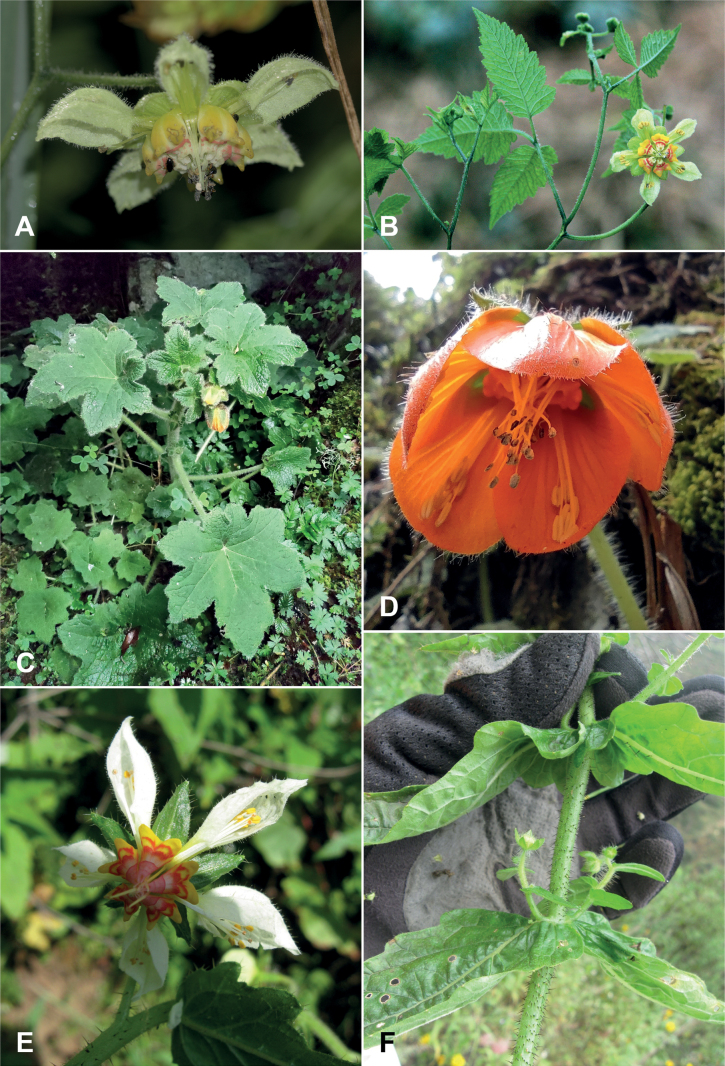
**A, B***Nasacolanii***C, D***Nasaferox***E, F***Nasahastata***A** flower of *N.colanii***B** flowering branch of *N.colanii***C** habit of *N.ferox***D** flower of *N.ferox***E** flower of *N.hastata***F** node with the characteristic, semiamplexicaulous leaves of *N.hastata*, Photo credits: **A, B** A. A. Wong Sato **C, D** E. Segovia **E, F** P. Gonzáles.

In the field, *Nasacolanii* differs from vegetatively similar *Nasaaequatoriana* by its much shorter, pale greenish-white petals (Fig. 1A, B). Additionally, the nectar scales of *N.colanii* are yellow and white with red transversal stripes, as in other species of the *Nasatriphylla* complex, but much paler with a very narrow red band only (Fig. 1A).

*Nasacolanii* was found on creek nanks in rocky soils in a cloud forest ecosystem located in the buffer zone of the Cordillera de Colán National Sanctuary (5°37'50.96"S, 78°15'20.84"W) at an elevation of 2605 m, near the Refugio Lechucita. This taxon had previously only been reported once in 1978 from the same region, possibly from the same locality (Rodríguez and Weigend 2006; Wong Sato et al. 2021). This species has probably not been collected since, due to its apparent narrow endemism and a lack of scientific exploration of this area (Rodríguez and Weigend 2006; C. Olivera, pers. comm., 2021).

#### Additional specimens examined.

**Peru. Amazonas**: Provincia Utcubamba, Distrito Cajaruro, buffer zone of the Cordillera de Colán National Sanctuary, ca. 2605 m, 5°37'50.96"S, 78°15'20.84"W 21 Dec 2019, *A.A. Wong Sato 53* (MOL).

#### Photographic record.

**Peru. Amazonas**: Provincia Utcubamba, Distrito Cajaruro. Buffer zone of the Cordillera de Colán National Sanctuary; observation by A. A. Wong Sato, 21 Dec 2019 (*Wong Sato 53*, MOL): https://www.inaturalist.org/observations/143281337.

### ﻿ Nasa ferox

Taxon classificationPlantaeCornalesLoasaceae

Weigend, Revista Peru. Biol. 13(1): 74 (2006).

B5218E08-AF4C-5982-BA3F-536C7228F0C7

Fig. 1C, D ; fig. 8 in Weigend 1996b

#### Type.

Ecuador. Azuay: Cantón Cuenca, Contrayerba, 3600–3800m, s.d., *F.C. Lehmann 7943* (holotype: US 00603973!; isotypes: F No. 578096!, K 000372883!).

Described only in 2000 (Weigend et al. 2006) from specimens collected by F. C. Lehmann probably in the 1880s (possibly May 1887, according to Cribb 2010, Lehmann was in Contrayerba at least twice). Specimens of this species were considered as belonging to *Loasaranunculifolia* Kunth (Urban and Gilg 1900) or *Loasapeltata* Urb. & Gilg (Weigend 1996b). The species was known with certainty only from the area of Contrayerba, in the province of Azuay close to the NW border of what is now Parque Nacional Cajas and had not been reported for ca. 130 years. Given the location of the park close to the city of Cuenca, and the fact that the important road 582 goes through the park makes it particularly surprising that the species has not been reported in such a long time, even more so if we consider the numerous botanical expeditions that have been carried out in the general region. New photographs uploaded by ES to iNaturalist in 2022 clearly show living plants of this species previously known only from dried specimens. Judging from the pictures now available, the species seems closely allied to *Nasajungiifolia* (Weigend) Weigend from just a little bit further south in Azuay, but differs from it in the smaller stature of the plants (20–70 cm, Fig. 1C) and the shorter, wider and fleshy, deep orange petals (Fig. 1D) (versus taller plants to >1 m in height and narrower, long acuminate, membranous, pale orange petals in *N.jungiifolia*). The habitat of the living plants of *N.ferox* is located in a rock outcrop at the foot of a vertical rocky cliff, with nearby pastures, some used for llama ranching. The substrate is covered by a dense layer of mosses along with succulent *Peperomia* spp. (Piperaceae), *Stellaria* spp. (Caryophyllaceae), *Oxalis* spp. (Oxalidaceae) and ferns. Tall shrubs and small trees such as *Polylepisreticulata*, *Gynoxys* spp. (Asteraceae), and an undetermined Asteraceae were the main woody species of this habitat. The slope in the site is low, about 30 degrees. *Nasaferox* is not an abundant species; only a very small population of about ten fertile plants growing in four spots near the borders of the rock zone could be found, with the plants growing always in sheltered places, in rock crevices, near big rocks or at the base of dense, taller, shrub aggregations. Some regeneration was observed at the end of the rain season in July, with a few seedlings growing among the moss carpets that covered the rocks. We also saw some infertile plants growing near the fertile ones in two of the spots. Footprints and dung from llamas and bovines on the trails nearby, show that livestock roam the area.

#### Additional specimens examined.

**Ecuador. Azuay**: Province unknown: „Andes of Ecuador“, *R. Pearce 1862* (K); Provincia Azuay, Cantón Cuenca, Reserva de la Biosfera Macizo del Cajas, 3835 m, 12 Jan 2022, *E. Segovia 3239-CMP40* (HA); Reserva de la Biosfera Macizo del Cajas, 3823 m, 07 Jul 2022, *E. Segovia 4890-CMP40* (HA).

#### Photographic records.

**Ecuador. Azuay**: Cantón Cuenca, Reserva de la Biosfera Macizo del Cajas, 2.78118S, 79.26592W, 3835 m, E. Segovia, Jan 2022, https://www.inaturalist.org/observations/105051734 (https://www.gbif.org/occurrence/3465963568); Reserva de la Biosfera Macizo del Cajas, 2.778691S, 79.266028W, 3825 m, K. Montesinos, 21 May 2022, https://www.inaturalist.org/observations/145275636 (https://www.gbif.org/occurrence/4011672795); Reserva de la Biosfera Macizo del Cajas, 2.782492S, 79.267025W, 3823 m, E. Segovia, Jul 2022, https://www.inaturalist.org/observations/125099917 (https://www.gbif.org/occurrence/3858810457); Reserva de la Biosfera Macizo del Cajas, 2.734655S, 79.259702W, G. Normand, Apr 2022, https://www.inaturalist.org/observations/112870994 (https://www.gbif.org/occurrence/3764320941).

### ﻿ Nasa hastata

Taxon classificationPlantaeCornalesLoasaceae

(Killip) Weigend, T.Henning & R.H.Acuña comb. nov.

3F6D24F5-81F1-5450-8EC0-BA10A9B1E326

urn:lsid:ipni.org:names:77322127-1

Fig. 1E, F ; also see Weigend and Rodríguez 2003: fig. 18A–F

Loasa hastata Killip, J. Wash. Acad. Sci. 18(4): 92 (1928). Basionym. Type: Peru. Lima. Provincia Huarochirí, Matucana, 2500 m, Apr–May 1922, *J.F. Macbride & W. Featherstone 416* (holotype: F! [acc. # 516950!]; isotype: US! [00115210, acc. # 1230340]).

#### Type.

Based on *Loasahastata* Killip.

Like *N.solaria* (J.F.Macbr.) Weigend (see below), this species was collected by J. F. Macbride and W. Featherstone in what is now the province of Huarochirí in the department of Lima. No subsequent collections were known of this species, and it was considered extinct in the wild (Weigend and Rodríguez 2003). The affinities of this morphologically distinctive Central Peruvian endemic remain obscure. Its leaf shape is unique in *Nasa* and the nectar scales are also very distinctive (Fig. 1E, F). Weigend and Rodríguez (2003) considered it as part of the *Nasastuebeliana* (Urb. and Gilg) Weigend species group, a group that is otherwise most likely monophyletic and mostly restricted to the southern half of the AHZ. Recent field studies by PG yielded new observations of this taxon from the districts of Arahuay and Santa Rosa de Quives in the province of Canta, showing that it is still present in the department of Lima.

In Arahuay, this species is restricted to a narrow altitudinal range between 2450 and 2500 m on shrubland. Only one sterile individual was recorded and photographed in April 2009. Two years later (2011) a small population of five flowering individuals was encountered and photographed by Elizabeth Gonzáles, the sister of PG. Another expedition to the site in April 2015 by PG and Tim Böhnert (Bonn) yielded only two sterile individuals. In Santa Rosa de Quives, PG and his colleague Eduardo Navarro walked a 500 m trail collecting plants across the shrubland for four hours and only recorded a single individual of *Nasahastata*. The two localities are only 7 km apart and are separated by a mountain ridge that reaches 3,500 m.

#### Note.

For obvious reasons iNaturalist limits the designation of taxon names to scientific names from external curated data sources such as IPNI (ipni.org). This helpful functionality revealed a nomenclatural issue with the name *Nasahastata*, which was not available. Instead only the basionym “*Loasahastata*” could be found in IPNI, together with the remark that due to an incorrect citation of the basionym reference, *Nasahastata* was a name not validly published which had been used in previous publications (Weigend in Weigend 1998: 164; Weigend and Rodríguez 2003: 377; Weigend in Weigend et al. 2006: 75). The nomenclatural problem is solved here by our validation of the combination.

This is a nice example of how meaningful linkage of individual databases not only offers quickly accessible information in a convenient form. It provides different dimensions of error avoidance by ensuring the correct spelling of names and authorities, but also revealing profound nomenclatural issues as in the present case.

#### Additional specimens examined.

**Peru. Lima**: Provincia Canta, Distrito Arahuay, Arahuay y alrededores, matorral, 2450 m, 11°34'13.05"S, 76°42'12.23"W, 28 Apr 2011, *P. Gonzáles et al. 1469* (USM); Distrito Santa Rosa de Quives, a 3.5 km de Pichu Pichu, matorral dominado por *Jungiaamplistipula* (Asteraceae) y *Barnadesiadombeyana*, 2165–2363 m, 11°34'13.05"S, 76°42'12.23"W, 6 Jun 2012, *P. Gonzáles & E. Navarro 1873* (USM [acc. # 275320]); *Unknown 625* (USM [acc # 174383]).

#### Photographic record.

**Peru. Lima**: Provincia Canta, Arahuay, P.Gonzáles, 30 Apr 2009, https://www.inaturalist.org/observations/100632141; Arahuay, 28 Apr 2011 (*Gonzáles et al. 1469*, USM), https://www.inaturalist.org/observations/139056447; Santa Rosa de Quives, 6 Jun 2012, (*Gonzáles and Navarro 1873*, USM acc. # 275320), https://www.inaturalist.org/observations/139059041.

### ﻿ Nasa humboldtiana (Urb. & Gilg) Weigend subsp. humboldtiana

Taxon classificationPlantaeCornalesLoasaceae

6F8A1471-103E-5338-8C3F-F603170FC9CC

Fig. 2A, B

Loasa humboldtiana Urg. & Gilg, Nova Acta Acad. Caes. Leop.-Carol. German. Nat. Cur. 76: 240, pl. 5, fig. 40 (1900). Type. Ecuador. Chimborazo: Cantón Chunchi?, in andibus ecuadorensibus, Llalla, Aug 1859, *R. Spruce 6002* (lectotype, designated in Weigend 1996a: 232: P! [00123885]; isolectotypes: B[destroyed, F Neg No. 10196!], BM! [BM000021453], E! [E00085319], F! [acc. #1540659], GH! [00076022], GOET!, K! [K000372786, K000372787, K000372788], M! [0113254], OXF!, P [P02273159] S! [acc. # S-R-8215], W! [0053328, 1889-0113217]).

#### Type.

Based on *Loasahumboldtiana* Urb. & Gilg.

**Figure 2.**
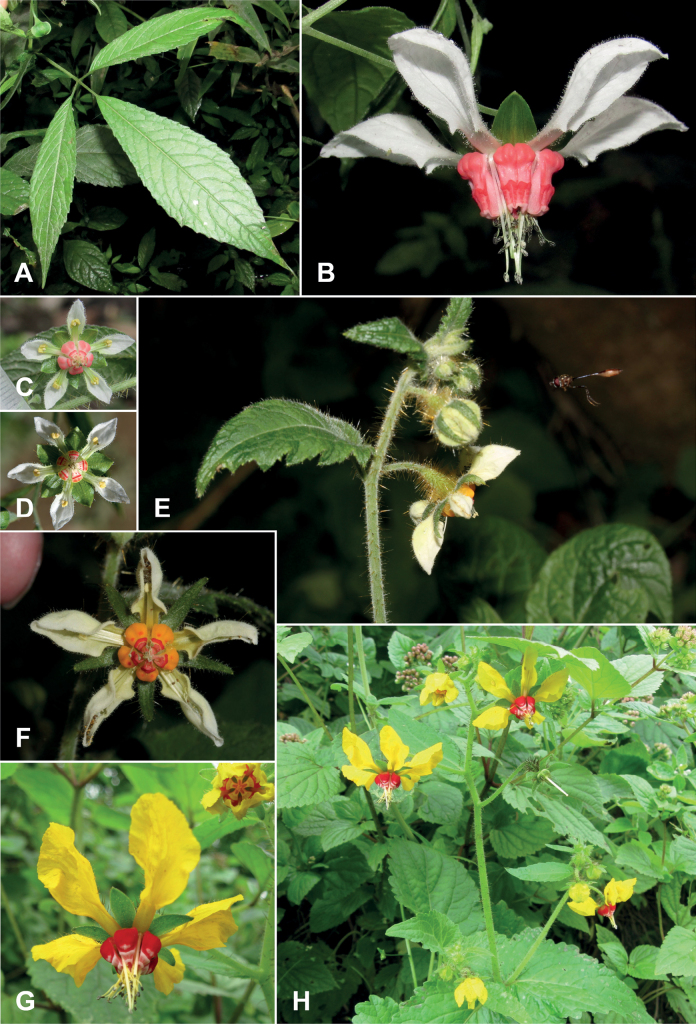
**A–D***Nasahumboldtiana***E, F***Nasaramirezii***G, H***Nasasolaria***A** trifoliolate leaf of N.humboldtianasubsp.humboldtiana**B** flower of N.humboldtianasubsp.humboldtiana**C** flower of N.humboldtianacf.subsp.obliqua from Chimborazo/Ecuador **D** flower of N.humboldtianasubsp.obliqua from Cajamarca/Peru **E** inflorescence of *N.ramirezii* with putative pollinator **F** flower of *N.ramirezii***G** flower of *N.solaria***H** habit of *N.solaria*. Photo credits: **A–C** X. Cornejo **D** T. Henning **E, F**: R. Ripley **G, H** P. Gonzáles.

*Nasahumboldtiana* belongs to the *Nasatriphylla* (Juss.) Weigend complex (Dostert and Weigend 1999), a natural group of herbaceous annuals found in the undergrowth of humid forest and disturbed sites in the northern Andes and Central America. This group is characterized by compound leaves (Fig. 2A), a trait that is very rare in *Nasa* (Acuña-Castillo et al. 2021). The highest number of species in this complex is in the AHZ, only two out of 24 taxa (including subspecies) grow exclusively outside this region (Dostert and Weigend 1999).

Nasa (Loasa) humboldtiana has been very poorly understood since its original description, with only two known collections from the 19^th^ century (Weigend 1996a). In recent years, extensive field studies yielded several new taxa closely allied to *Nasahumboldtiana*. In a synopsis of the *Nasatriphylla* complex (Dostert and Weigend 1999), *N.humboldtiana* was expanded to include three additional infraspecific taxa namely subsp. roseoalba (Weigend) Dostert (originally described as a distinct species, *Loasaroseoalba* Weigend: Weigend 1996a), subsp. tricolor Dostert & Weigend and subsp. obliqua Dostert & Weigend. Later, targeted field work led to the discovery of two additional subspecies. Nasahumboldtianasubsp.subtrifoliataWeigend & T.Henning andsubsp.glandulifera Weigend & T.Henning are endemic to small forest remnants in northern Peru and under imminent threat of extinction (Henning and Weigend 2009).

Nasa (Loasa) humboldtiana was described in 1900 (Urban and Gilg 1900), and the most recent herbarium collection by Richard Spruce dated back to Aug. 1859 from what today is the southern part of the province of Chimborazo, Ecuador (Spruce 1908 p. 222 & 249–250). Nasahumboldtianasubsp.humboldtiana differs most notably from the other subspecies by its remarkably different nectar scales, with a rectangular (instead of tapering) scale neck (Henning and Weigend 2009). In 2021, one of us (XC) collected material clearly referable to the typical subspecies humboldtiana in a ravine within a conserved remnant of montane Andean forest in El Corazón, a private property located in the province of Chimborazo (Fig. 2A, B), ca. 30 km straight line from the type locality of this taxon, which signifies the rediscovery of the taxon after 162 years. The same population, represented by fewer than five individuals, has been observed in the field flowering at least twice (XC), most recently in August 2022.

#### Additional specimens examined.

**Ecuador. Chimborazo**: Cantón Pallatanga, Reserva El Corazón, a montane Andean forest, ca. 2700 m, 2°3'S, 78°54'W, 10 Jul 2021, *X. Cornejo & J. Josse 9388* (GUAY).

### ﻿ Nasa humboldtiana (Urb. & Gilg) Weigend subsp. obliqua

Taxon classificationPlantaeCornalesLoasaceae

Weigend, Revista Peru. Biol. 13(1): 75 (2006).

47F2DEAE-C631-5FE4-A2E8-3771BC67D276

Fig. 2C, D

#### Type.

Peru. Cajamarca: Provincia Hualgayoc [Prov. Santa Cruz], Monte Seco, 1800 m, *J. Soukup 3826* (holotype: US! [00604255, acc. # 1985252]) .

Collected in the Reserva El Corazón, but in a semi open forest/grassland ecosystem with scattered native tree remnants adjacent to the forest where Nasahumboldtianasubsp.humboldtiana occurs, and on the same day, XC also collected and photographed another subspecies of *Nasahumboldtiana*, which is here tentatively assigned to N.humboldtianacf.subsp.obliqua (Fig. 2C). This taxon had previously only been reported from a single area in northern Peru, namely the Prov. Santa Cruz in the department of Cajamarca (Fig. 2D). The new record in Ecuador lies ca. 550 km north of the original collection on the western slope of the Peruvian Andes, and additional studies are required to confirm its identity. It may also represent a separate and novel taxon or unusually small-flowered plants of the widespread SW Ecuadorean endemic Nasahumboldtianasubsp.roseoalba, whose type locality is near Chillanes (Bolívar, Ecuador) ca. 20 km to the northwest. This taxon was even collected previously in El Corazón in sterile condition in Feb. 2017 (*R. Acuña & D. Guilcapi 1725*, QCA, BONN). The leaflet texture and leaflet base shape of the newly collected plants appear to be somewhat intermediate between more typical N.humboldtianasubsp.roseoalba and N.humboldtianasubsp.obliqua.

#### Additional specimens examined.

**Peru. Cajamarca**: Provincia Santa Cruz, Monte Seco, 1500 m, *N. Dostert 98/154* (CPUN, F, M, USM); La Florida, above Monteseco, 1200–1500 m, 5 May 2003, *M. Weigend et al. 7554* (B, HUT, USM, M); Near Agua Azul, 5 May 2003, *Weigend et al. 7569* (B, HUT, M, USM); **Ecuador. Chimborazo**: Reserva El Corazón, a montane Andean forest, ca. 2700 m, 2°03'S, 78°54'W, 10 Jul 2021, *X. Cornejo & J. Josse 9389* (GUAY).

### ﻿ Nasa ramirezii

Taxon classificationPlantaeCornalesLoasaceae

(Weigend) Weigend, Revista Peru Biol. 13(1): 80 (2006).

D099AB3A-CE53-5239-AC8E-4CBD4722B59B

Fig. 2E, F ; fig. 6 in Weigend 1996a

Loasa ramirezii Weigend, Sendtnera 3: 231 (1996). Type. Colombia. Nariño: Municipio Tangua, 5 km S of Tangua [vertiente al otro lado del valle sur de Tangua], 2600 m, *M. Weigend & B.R. Ramírez 3280* (holotype: M! [M-0113266]; isotypes: COL!, PSO! [PSO0000004, PSO0000005]).

#### Type.

Based on *Loasaramirezii* Weigend.

The rediscovery of *Nasaramirezii* is here reported for Ecuador only. It was described in 1996 based on a collection made in the southern Colombian department Nariño by MW and cultivated plants thereof. From Ecuador, however, only a small number of very old specimens was known which all lack proper locality information. These four collections all go back to the 19^th^ century and could only tentatively be assigned to this species, whose occurrence was only secured from a few small patches in southern Colombia so far. Until now, this taxon could only be assumed to occur in northern Ecuador, and given the widespread habitat destruction in this region, it was suspected that it might be extinct there. Two recent observations uploaded to iNaturalist revealed the first photographs of living plants from Ecuador and the first exact locality information. The taxon is apparently restricted to a small area in the province of Imbabura and has been repeatedly observed in the Conrayaro forest.

Two independent observations have been recorded from the same area by Ruth Ripley in March 2018 and Mony León in April 2023, respectively. The former shared the following detailed occurrence data with us: flowering plants of *Nasaramirezii* were found on the path to the Cascada de Conrayare, in San Alfonso de Iruguincho, in Timbuyacu, to the southwest of Cerro de Añaburo at elevations between 2700–3000 m. This is located in San Miguel de Urcuquí County, Imbabura Province in NW Ecuador. The area is dominated by Andean forest and some common angiosperms include for example *Barnadesia* sp., *Bomarea* sp. (Alstroemeriaceae), Ericaceae, *Geranium* sp. (Geraniaceae), Melastomataceae, *Oxalis* sp., *Peperomia* sp., *Phyllanthus* sp. (Phyllanthaceae), *Salvia* sp. (Lamiaceae) and *Siparuna* sp. (Siparunaceae).

#### Additional specimens examined.

**Colombia. Valle de Cauca**: Popayan, Western slopes of the Sotara Volcanoe, 2400 m, *Lehmann 6205* (K); **Nariño**: Tangua, Tapialquer, 2250–2500 m, *B.R. Ramirez s.n.* (PSO); 5km south of Tangua in a coffee plantation, 2600 m, *M. Weigend 3280 & B.R. Ramirez* (M, COL, PSO); Tajumbina, Mpio de la Cruz, 2630 m, *Buenavides s.n.* (PSO); Mpio Consaca, Mpio de Coriaco, 1820 m, *Ramirez s.n.* (PSO); Mpio de Consaca, Seccion de Coriaco, 1820 m, *Guarin 407* (PSO). **Ecuador. Province unknown**: “Andes of Quito”, *Jameson 79* (K); “Andes of Cuenca at 10,000 feet in woods, July 1840” *Jameson 289* (K); Without locality, *Jameson s.n.* anno 1840 (E); “Loasa sp. Nova de Huayaquil [Guayaquil]”, *Ruiz & Pavón s.n.*, leg. Tafalla (BM, G).

#### Photographic records.

**Ecuador. Imbabura**: San Miguel de Urcuquí, Conrayaro forest, 0.427288S, 78.270863W, 14 Mar 2018, R. Ripley https://www.inaturalist.org/observations/12800711 (https://www.gbif.org/occurrence/3466042315); W 78.278315, 0.436863, 2 Apr 2023, M. León https://www.inaturalist.org/observations/153269256.

### ﻿ Nasa solaria

Taxon classificationPlantaeCornalesLoasaceae

(J.F.Macbr.) Weigend, Revista Peru. Biol. 13(1): 80 (-81) (2006)

80D4FA8B-9E85-5E2C-827E-1B00D8FA9EAA

Fig. 2G, H

Loasa solaria J.F.Macbr, Publ. Field Mus. Nat. Hist., Bot. Ser. 13(4): 163 (1941). Type. Peru. Lima: Provincia Huarochirí, San Miguel de Viso, ca. 2750 m, May 1922, *J.F. Macbride & W. Featherstone 577* (holotype: F! [acc. # 517105]; isotype: US! [00115216, acc. # 1230343]).

#### Type.

Based on Loasasolaria J.F.Macbr.

Nine species of *Loasa* originally described in the Flora of Peru (Macbride 1941) are today included in *Nasa*. All of these are still considered distinctive and accepted as good species (Weigend et al. 2006). Although all were described from relatively few specimens (some collected by Macbride himself), seven have been rediscovered and studied in the wild and/or in cultivation in the past decades. Two species, however, still remain unknown (or almost so) in the wild: *Nasaaspiazui* from Junín (collected by A. Weberbauer) and *N.solaria* from Lima (collected by J. F. Macbride and W. Featherstone). *Nasasolaria* is the only species in the genus with the combination of entire, shallowly lobed leaves and flowers with deep yellow petals and bright red nectar scales and cannot be confused with any other species (Fig. 2G, H). *Nasasolaria* is morphologically quite aberrant in the genus, and this has rendered a morphological placement difficult. Plastid DNA (obtained after the rediscovery of the species) seems to indicate it could be allied to the morphologically plesiomorphic *Nasapoissoniana* (Urb. & Gilg) Weigend species group (Acuña-Castillo et al. 2021). The original collection of *Nasasolaria* came from the department of Lima, province Huarochirí – and despite its proximity to the national capital, about 80 km, it has been recovered only once in the last century in this area. This and a second collection from the department of Lima in 1998, as well as two earlier collections from the neighbouring department of Ancash, remained undiscovered in the herbarium in Lima (USM) until a targeted search was conducted after the species was recently rediscovered and the first photos of living plants reached us. The area of the original collection has been subject to massive human intervention and land use change, possibly leading to local extinction. Recently, there have been several new collections of this species from the Province of Huarochirí, where the original material came from and the neighbouring Provinces Canta and Huaral, confirming that the species remains rare, but is still present in the area. This species is restricted to an elevational range of ca. 1000 m between 2800 and 3600 m; currently, two populations are known from the undergrowth of relict forests. The other two known localities, Carhua in Prov. Canta and Rupac in the Prov. Huaral, are in shrubland, where very scattered, small trees of *Myrcianthesquinqueloba* and *Escalloniaresinosa* can still be found, indicating that these areas were previously covered with forest, but that despite deforestation, *Nasasolaria* still grows there. In the forests of Zárate, Prov. Huarochirí, only two individuals were found. In the forests of Huarimayo, Canta, four populations separated by ca. 300 m in a linear transect were found between the years 2015 and 2022 with a total of only seven individuals. At Rupac, Huaral, one population was recorded with two individuals in April 2018 and five plants in May 2018.

#### Additional specimens examined.

**Peru. Ancash**: Provincia Bolognesi, Acas, monte bajo, borde de chacra, 3600 m, 16 Jun 1969, *E. Cerrate 7463* (USM 284507); Subida de la Rinconada a la cumbre, camino de Ocros, monte bajo, 3000 m, 2 May 1977, *E. Cerrate 6646* (USM 271500); **Lima**: Provincia Canta, Carhua, en la carretera hacia Pariamarca, ladera arcillosa con arbustos perennifolios, 3300 m, 3 May 1998, *G. Segovia 4756* (USM 277067); Distrito San Buenaventura, San José, justo en el límite con Huamantanga, bosque relicto, 2800–3000 m, 11°30'29.59"S, 76°42'35.98"W, 30–31 May 2015, *P. Gonzáles et al. 3773* (USM 290273); bosque relicto de Huarimayo, bosque relicto, 2877 m, 11°30'29.59"S, 76°42'35.98"W, 12 May 2022, *P. Gonzáles et al. 10470* (USM); Arriba de San Bartolomé, Monte Zárate, 2900–3000 m, 29 May 1954. *R. Ferreyra 9712* (USM 28005); San Bartolomé, Monte de Zárate, matorral y relicto de bosque dominado por *Oreopanax*, *Myrcianthes* entre otros, 1440–3550 m, 11°55'46.25"S, 76°29'36.55"W, 24–26 [25] Apr 2009, *P. Gonzáles et al. 492* (USM 256800); Provincia Huaral, Distrito. Atavillos Bajo, Pampas, subida a Rupac, ladera con suelo franco-arcilloso, matorral, 3033–3509 m, 11°19'23.00"S, 76°46'52.97"W, 15 Apr 2018, *A. Cano et al. 22677* (USM 327563); Pampas, en las cercanías al centro poblado y camino al complejo arqueológico de Rupac, ladera con afloramiento rocoso suelo franco-arcilloso a franco-arenoso, matorral, 3033–3099 m, 11°19'23.00"S, 76°46'52.97"W, 7 [May] Jun 2018, *A. Cano et al. 22723* (USM 327614).

#### Photographic record.

**Peru. Lima**: Provincia Canta, Bosque de Huarimayo, W. Aparco, 31 May 2015 (*P. Gonzáles et al. 3773*, USM acc. # 290273), https://www.inaturalist.org/observations/139042423 (https://www.gbif.org/occurrence/3947631714); Provincia Huarochirí, Bosque de Zárate, 25 Apr 2009 (*P. Gonzáles et al. 492*, USM acc. # 256800), https://www.inaturalist.org/observations/118647914 (https://www.gbif.org/occurrence/3802749525); P. Gonzáles, 27 May 2019, https://www.inaturalist.org/observations/118647465 (https://www.gbif.org/occurrence/3802781447); Provincia Huaral, Rupac, P. Gonzáles, 15 Apr 2018 (*Cano et al. 22677*, USM acc. # 327563), https://www.inaturalist.org/observations/139044179 (https://www.gbif.org/occurrence/3947206738); P. Gonzáles, 7 May 2018 (*Cano et al. 22723*, USM acc. # 327614), https://www.inaturalist.org/observations/100467047 (https://www.gbif.org/occurrence/3416222423).

## ﻿Discussion

Ongoing documentation of biodiversity by trained botanists continues to yield important species records – even in, by tropical South American standards, relatively well sampled countries such as Ecuador and highly accessible regions such as the department of Lima in Peru. The rediscovery of Nasahumboldtianasubsp.humboldtiana after more than 160 years, the tentative range extension of Nasahumboldtianasubsp.obliqua and the rediscoveries of *Nasasolaria*, and especially *Nasahastata*, after nearly 100 years, near Lima, are typical examples of these crucial contributions to our understanding of species ranges and conservation.

However, many non-botanists get out into the field, lacking the requisite permits, training and ambition to prepare specimens and deposit them in a public repository. Photographic documentation of biodiversity, however, is a pastime for many and coincidentally may lead to highly relevant taxon records, particularly if they end up being uploaded into public databases. The records on iNaturalist can be studied by professional taxonomists, who are able to provide accurate determinations, or even recognize undescribed taxa, provided that the images uploaded show diagnostic traits of a taxon in sufficient detail. These digital records efficiently complement the records obtained from scientific collections, such as herbaria or museums, and we agree that the inclusion of these records into GBIF is appropriate.

Of course, where both data sources, photographic records and physical herbarium specimens, are linked, this creates a tremendous added value (Heberling and Isaac 2018). Purely digital occurrence records should not be understood as a substitute for physical specimens (Daru and Rodriguez 2023), but due to a range of reasons (see above) photographic records are often created under conditions where the preparation of physical specimens would be impossible or illegal. Conversely, specimens collected in the field should ideally be supplemented by, for example, an iNaturalist record, and vice versa, provided that collecting a specimen is permitted and justifiable.

Information about a species´ geographic range and possibly even abundance can be gathered from specimens kept in scientific collections, taxonomic revisions, field guides and similar works, but this data is often diffuse due to the long time period aggregated into the characterisation of range and abundance. iNaturalist can provide a sharper picture in time and space, since it documents taxa from a specific place and time and usually soon after the observation, providing a much more current view of occurrences. This has helped us and our colleagues to locate areas where the chances of finding and collecting a species are high, saving time and resources during field research. Due to these advantages, data obtained from iNaturalist are increasingly included in professional systematic, floristic, ecological, and conservation studies (Atha et al. 2020; Soteropoulos et al. 2021; Iwanycki Ahlstrand et al. 2022; Smith et al. 2022). Critically, and as in the case of this study, iNaturalist’s data has also led to the rediscovery of populations of taxa considered as extinct for many years as is the case of *Gasteranthusextinctus* L.E.Skog & L.P.Kvist (Gesneriaceae) by Pitman et al. (2022), *Scarabaeussevoistra* Alluaud, 1902 (Coleoptera: Scarabaeinae) by Deschodt et al. (2021), and *Tipulodesannae* Przybyłowicz, 2003 (Lepidoptera, Erebidae) by Alzate-Cano and Hurtado-Pimienta (2021). Hopefully, as more scientists and members of the public contribute to the database, and more professionals get involved in the curation (Callaghan et al. 2022), more undescribed or “long lost” taxa will be found. Our examples of the rediscovery of *Nasaferox* after 130 years and *Nasahastata* after 100 years, both “found” on iNaturalist underscore this point.

Conservation in the long term is only possible with the involvement of the human population at large and most importantly of the local communities. Biological education programs and the easy access to tools that permit the nature enthusiasts to document their encounters with nature can raise awareness and attract more people into investigating their natural environment (Echeverria et al. 2021), increasing their appreciation and understanding of biodiversity and ecology. iNaturalist.org has simultaneously become an important contributor to the knowledge of biodiversity and a successful tool for public engagement (Aristeidou et al. 2021).

## Supplementary Material

XML Treatment for Nasa colanii

XML Treatment for Nasa ferox

XML Treatment for Nasa hastata

XML Treatment for Nasa humboldtiana (Urb. & Gilg) Weigend subsp. humboldtiana

XML Treatment for Nasa humboldtiana (Urb. & Gilg) Weigend subsp. obliqua

XML Treatment for Nasa ramirezii

XML Treatment for Nasa solaria

## ﻿Additional information

### Author ORCIDs

Tilo Henning https://orcid.org/0000-0003-1929-3264

Rafael Acuña-Castillo https://orcid.org/0000-0002-8568-8566

Xavier Cornejo https://orcid.org/0000-0002-4081-4047

Paúl Gonzáles https://orcid.org/0000-0002-6799-376X

Akira Armando Wong Sato https://orcid.org/0000-0002-3617-8121

Maximilian Weigend https://orcid.org/0000-0003-0813-6650

### Data availability

All of the data that support the findings of this study are available in the main text or Supplementary Information.
